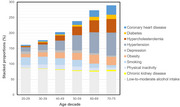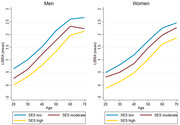# LIfestyle for BRAin health (LIBRA) and cognitive functioning from young to old adulthood: Results of the German National Cohort (NAKO)

**DOI:** 10.1002/alz70860_106257

**Published:** 2025-12-23

**Authors:** Susanne Roehr, Felix Georg Wittmann, Melanie Luppa, Sebastian Köhler, Kay Deckers, Colin Rosenau, Luca Kleineidam, Michael Wagner, Klaus Berger, Alexander Pabst, Steffi G. Riedel‐Heller

**Affiliations:** ^1^ Global Brain Health Institute (GBHI), Trinity College Dublin, Dublin, Ireland; ^2^ Institute of Social Medicine, Occupational Health and Public Health (ISAP), Medical Faculty, University of Leipzig, Leipzig, Saxony, Germany; ^3^ Institute of Social Medicine, Occupational Health and Public Health (ISAP), Medical Faculty, University of Leipzig, Leipzig, Germany; ^4^ Alzheimer Center Limburg, Mental Health and Neuroscience Research Institute, Maastricht University, Maastricht, Netherlands; ^5^ Department of Psychiatry and Neuropsychology, Alzheimer Centrum Limburg, Mental Health and Neuroscience Research Institute (MHeNs), Maastricht University, Maastricht, Netherlands; ^6^ Department of Psychiatry and Neuropsychology, Maastricht University Medical Center+, Maastricht, Netherlands; ^7^ Alzheimer Center Limburg, School for Mental Health and Neuroscience, Maastricht University, Maastricht, Netherlands; ^8^ Department of Neurodegenerative Diseases and Geriatric Psychiatry, University of Bonn Medical Center, Bonn, Germany; ^9^ German Center for Neurodegenerative Diseases (DZNE), Bonn, Germany; ^10^ Institute of Epidemiology and Social Medicine, University of Muenster, Muenster, Germany

## Abstract

**Background:**

The LIfestyle for BRAin Health (LIBRA) index is a well‐validated tool for assessing modifiable dementia risk in midlife and older adults. Less is known about LIBRA in younger adults. Thus, we investigated the occurrence of LIBRA factors and associations between the LIBRA index and cognitive functioning across adulthood, spanning ages 20 to 75. We considered variations by age decade, sex, and socioeconomic status (SES).

**Method:**

The data source was the population‐based mega‐cohort “German National Cohort” (NAKO). Proportions and Cochran‐Armitage trend tests were calculated for 10 out of 12 LIBRA factors (coronary heart disease, diabetes, hypercholesterolaemia, hypertension, depression, obesity, smoking, physical inactivity, chronic kidney disease, and low‐to‐moderate alcohol consumption; no information available on healthy diet and high cognitive activity). Cluster‐adjusted (for study sites) linear regression analysis was used to assess associations of LIBRA scores and cognitive functioning (composite neuropsychological test score), adjusted for age, age^2^, sex, education, SES, employment status, marital status, household size, migration status, and German language proficiency, for the total and stratified samples.

**Result:**

The analytical sample of 149,948 participants had a mean age of 50.1 (13.6) years; 50.4% were women, and education levels were high (56.2% had tertiary education). Behavioural risk factors (smoking, physical inactivity, depression) occurred more frequently in younger adults, while risk factors related to vascular health conditions (hypertension, diabetes, coronary heart disease) were more common in older adults (Figure 1). Higher LIBRA scores were consistently associated with lower cognitive functioning across adulthood. An SES gradient in LIBRA scores was observed across age decades and sexes (Figure 2). Men had lower LIBRA scores, but associations with cognitive functioning were more pronounced in women.

**Conclusion:**

Our findings provide novel evidence suggesting that LIBRA is a useful tool in younger adulthood (20–39 years), a group still neglected in dementia risk research. Modifiable risk factors were already frequent in this age range and associated with lower cognitive functioning. The observed sex/gender and socioeconomic disparities indicate compounded disadvantages faced by lower SES groups and women, emphasising the need for tailored, ideally early interventions possibly targeting behavioural risk factors. Longitudinal studies could help to disentangle life‐course dynamics of LIBRA factors and cognitive functioning.